# The simultaneous effects of pharmaceutical policies from payers’ and patients’ perspectives: Italy as a case study

**DOI:** 10.1007/s10198-015-0739-0

**Published:** 2015-10-27

**Authors:** Patrizio Armeni, Claudio Jommi, Monica Otto

**Affiliations:** 1 CERGAS Bocconi, Via Sarfatti, 25, 20136 Milan, Italy; 2 CERGAS Bocconi, Università del Piemonte Orientale, Largo Donegani, 2/3, 28100 Novara, Italy

**Keywords:** Pharmaceutical policies, Patients’ perspective, Mediated effects, Impact evaluation, I1, H5, H75

## Abstract

**Objectives:**

This paper aims at covering a literature gap on the effects of copayments, prescription quotas and therapeutic reference pricing on public and private expenditures and volumes (1) When these policies are implemented in different areas at different times, (2) estimating their impact in the short and long run, (3) assessing the extent to which these impacts are interdependent, (4) scrutinising the extent to which the effects are mediated by prescribers’ and patients’ behaviours.

**Methods:**

Monthly regional data on pharmaceutical expenditures, volumes and policies in Italy from 2000 to 2014 are analysed using a difference-in-differences model enriched to capture short- versus long-term effects and simultaneous and interactive effects. Sobel–Goodman test and bootstrap analyses were used to test for mediation.

**Results:**

The three policies have different short- and long-run effects. Interactions support the hypothesis of reinforcing effects. Behavioural reactions to policies such as reducing the demand or total per capita expenditures mediate the impact of policies, thus explaining the different effects between the short and long term.

**Conclusions:**

Evidence on the impact over time of regional policies diversely introduced in different times have important policy implications. First, pharmaceutical policies interact with each other, and the combined effect may be different from what we would expect from the sum of each single policy. Hence, policymakers should be very careful in designing mixed policies for their unexpected combined effects. Second, the impact of policies tends to reduce over time. If longer-term impact is desired, it would be appropriate to introduce some adjustments over time. Third, policies have multiple effects, and this should be considered when they are designed. Finally, pharmaceutical policies may have an unintended impact on health and health care.

## Introduction

In the last 20 years, pharmaceutical policies have been mostly driven by the cost-containment imperative. Therefore, a primary research target has been the impact of pharmaceutical policies on public expenditures [1–5], prices [3, 6, 7] and demand/quantities [4, 5, 8–12].

Literature on the impact of pharmaceutical policies has some limitations. First, most studies focused either on a single therapeutic class [8, 10–12] or on the effects of a single policy [1, 3, 9]. Second, the simultaneous impact of different policies [11] and their effects over time [2] have not been investigated. Third, when more dependent variables (e.g. drug volumes and expenditures) were scrutinised [4, 5, 7], they were independently analysed. Thus, despite evidence that pharmaceutical policies impact more than one variable, little is known about how these effects are interdependent, such as how much the reduction in public expenditures is caused by a fall in the demand for pharmaceuticals. The literature has investigated the effects of reference pricing [2, 7, 13, 14] and copayment [5, 11, 12, 15]. However, research has not considered regional policies together with the combined effects of different policies over time.

The aim of this paper is to fill these information gaps using Italy as a case study. First, we evaluate the simultaneous and interactive effects of three pharmaceutical policies on public and private retail drug spending and quantities, both in the short and long term. The policies include copayment, prescription quotas (i.e. binding prescription targets per therapeutic class) and therapeutic reference pricing (TRP) (i.e. using a reference price per therapeutic class and having patients cover the difference between prescription and reference price). Therapeutic reference pricing is based on a cluster for price comparison larger than in the most common reference pricing, which is generally only applied to the same molecule or molecule–package pair. Second, we tested a behavioural mediation hypothesis to assess whether and how the impact of these policies on public expenditures is related to (1) a change in volume, holding private expenditures constant (and estimating the effects of other mechanisms, such as pure price effects) and (2) a shift from public to private coverage, holding volumes constant.

Italy serves as an ideal case study, as a wide range of pharmaceutical policies have been autonomously applied by Italian regions. As a consequence, data are available on “treated” and “untreated” regions over different periods and with different combinations. As highlighted by other authors [5], Italy represents a natural experiment because policies are implemented by regional governments after the central government has approved drug marketing and regulated prices and reimbursements. Nonetheless, the impact of Italian regional policies on pharmaceutical expenditures, with the exception of copayment [5], has not been scrutinised. Previous studies have conducted descriptive analyses of policies [16, 17] and pharmaceutical expenditure trends [4, 17, 18].

### Background and hypotheses

Since 1992, pharmaceuticals have been a preferred target of cost-containment actions [17]. Centralised price cutting, discounts on list prices and drug delisting prevailed in the first 10 years. Cost-containment approach was strengthened with the introduction of a spending cap on drugs (set as a percentage of public health funds) that was enforced by law in 2001. Initially, general price cuts were applied to cover the deficit. Since 2007, the industry has been partially in charge of covering the deficit: each pharmaceutical company is given a budget based on the national drug budget for the current year and market shares in the previous year; if the actual drug spending is over the budget, each company will contribute to the payback in proportion to its actual revenue (compared with its budget). Reimbursement and ex-factory prices are simultaneously negotiated by the national drug agency and the relevant company. The main criteria used in negotiation are disease burden, place in therapy and availability of alternative treatments, risk–benefit profile, therapeutic added value and impact on the drug budget. For most new drugs, managed market (financial-based and outcome-based) contracts are agreed on; for some drugs, more than one contract is negotiated [19]. Finally, reference pricing for genericated molecules (molecules with at least one generic version available) was introduced nationwide in 2001 and applied to the same molecule–package pair. TRP, instead, was introduced as a policy option implementable by regional governments in 2006.

Despite price and reimbursement being managed at the central level, since 2002, regions have implemented diverse actions to face financial constraints. In fact, regional governments have become accountable for their health-care-spending deficits. As a consequence, they strengthened their cost-containment actions on pharmaceuticals [17]. Copayment, actions on prescribing behaviour, including prescription quotas, and TRP were introduced by various regions to curb the retail market, whereas drugs used in hospital settings have been affected by regional formularies and procurement policies [4, 20].

Copayment in Italy is active in two forms: as a prescription fee and as the spread on the reference price. The latter has been active since 2001 (and then optionally increased in level with TRP) and is more the effect of a policy than a policy itself. Copayment as prescription fee, instead, was first introduced by regional governments in 2002 and is the form considered in this study as “copayment” policy; in 2014, drugs were subject to copayment in 16 ut of 21 regions. The introduction of copayment produces a payment shift from third-party payers to patients and is expected to lower drug consumption, at least in the short term. Therefore, we expect a short-term decline in public expenditures and increase in private expenditures. In absolute terms, private expenditures are expected to be lower than public expenditures because of the drop in consumption. In other words, we expect consumption to mediate the effects of copayment on public expenditures. In the long term, patients and prescribers may adjust their initial choices and increase consumption, leading to a positive impact on both public and private expenditures.

Prescription quotas were first introduced in 2005. They refer to binding prescription targets that address general practitioners prescribing more genericated molecules within a certain therapeutic class (e.g. x Percentage of genericated statins over the total prescription of statin). These quotas are usually enforced by (regional) law and linked with sanctions/incentives for prescribers. For the most important retail therapeutic classes [e.g. hypertension drugs, statins, selective serotonin reuptake inhibitors (SSRIs) and proton pump inhibitors (PPIs)], quotas were applied in 13 regions in 2014. Prescription quotas are expected to shift prescriptions within a therapeutic class of drugs from expensive to less expensive, with an overall reduction in public expenditures. No effect is expected on volume unless cheaper drugs lead to increased consumption, and no effect is expected on private expenditures unless generic products are associated with generic molecules that involve copayment due to reference pricing. We expect that prescription quotas require behavioural adjustments by patients and prescribers, and we expect these adjustments to occur over the long term.

TRP was intended to reduce the expected impact of generic reference pricing, which enhances a prescription shift from genericated molecules to patent-protected drugs in the same therapeutic area, avoiding the application of generic reference pricing. As of 2014, TRP was applied only to PPIs in nine regions. In October 2007, around 1 year after its introduction, this policy option was abolished at the regional level for equity reasons, but the new regulation has not had any retroactive effect, such that regions that had already activated TRP were allowed to maintain it. In principle, TRP is expected to reduce public and increase private expenditures. However, in the absence of copayment and prescription quotas, TRP could have adverse effects on public expenditures. In fact, TRP reduces the perceived minimum price for both private and public payers and could stimulate a higher demand. Because the demand mechanism is behavioural, we expect the effect to occur in the long term.

An interaction of policies is expected to occur to the extent to which the mechanisms behind them are not independent. Depending on how their mechanisms interact, one policy can reinforce or hinder the effects of the other. We expect that the three policies reinforce each other in decreasing public expenditures. The simultaneous presence of copayment and prescription quotas should lead prescribers to reduce inappropriate prescriptions and to favour cheaper drugs. Similarly, copayment associated with TRP should orientate prescribing behaviour toward less expensive molecules. Finally, prescription quotas should mitigate the expected positive impact of TRP on volumes, and TRP may enhance a shift towards cheaper drugs that are produced by prescription quotas. However, interactive mechanisms may exceed the intended impact, generating less equity and possible undertreatment. This effect could be signalled by a shift from reimbursed to nonreimbursed drugs.

## Materials and methods

### Policy impact analysis applied to drugs

Following the aforementioned gaps in the literature, this paper aims to assess (1) the individual and interactive effects of the three pharmaceutical policies on public and private retail drug spending and volumes in the short and long term, and (2) the causal relationship among policies, prescription/consumption behaviours and both public and private expenditures (i.e. the extent to which the long-term effect on expenditures is mediated by behaviours). Several models have been used to evaluate policy impact [21, 22]. We employed an enriched difference-in-differences (DD) model that allows simultaneous estimation of the effects of three policies and their interactions. We first estimated the separate effects of policies on public expenditures, private expenditures and volumes. Then, we tested the hypothesis that the effects of pharmaceutical policies on public expenditures are mediated by prescription/consumption behaviours (i.e. a transmission mechanism). To test for robustness, we also tested for possible reverse-causality and feedback mechanisms by switching the mediator and the independent variable, which allowed us to rule out the alternative hypothesis of ambiguous causality on behaviours.

### Variables under consideration

**Fig. 1:**
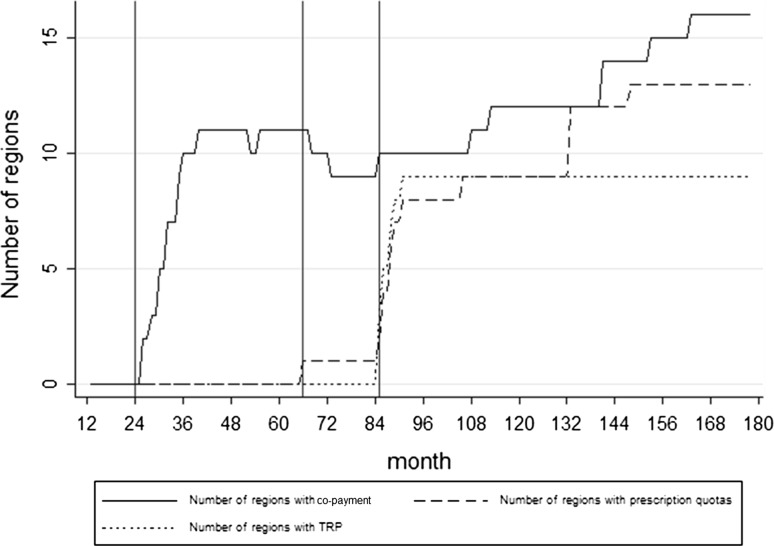
Number of regions by month and policy (21 regions, 2000–2014). *Note*: Month 1 is January 2000, and month 180 is December 2014

**Fig. 2:**
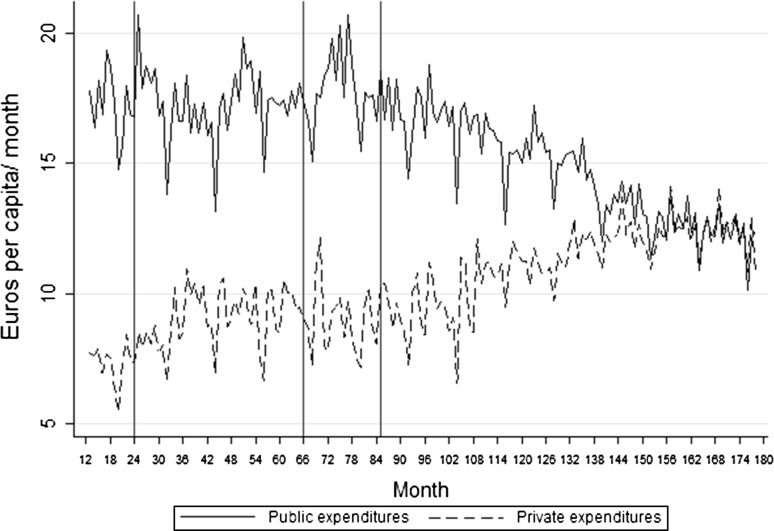
Monthly public and private pharmaceutical expenditures in Italy (2000–2014)—euros per capita. Month 1 is January 2000, and month 180 is December 2014. The *three vertical lines* show the first month in which copayment, prescription quotas and therapeutic reference pricing (TRP), respectively, from *left* to *right*, were first introduced

Policies considered and their period of activation in every region are reported in Table 1. Figure 1, on the other hand, summarised the number of regions in which each policy was active in every period. Included variables, their measurement and relevant sources are summarised in Table 2. Dependent variables included monthly per capita public and private expenditures and volumes for partially and fully reimbursed as well as nonreimbursed retail drugs. Volumes were also used as mediators when testing for a behavioural transmission mechanism. The independent variable matrix included policies in the short term (i.e. introduced within 6 months) and long term (i.e. introduced at least 6 months earlier). We also included a set of control variables. Public and private expenditures are influenced by several factors other than policies. For example, in an investigation on the impact of copayment on drugs in Italy, Fiorio and Siciliani [5] included control variables and used a fixed-effects model (using the first-difference approach, equivalent to a fixed-effects model, since the number of periods was 2); the authors included a dummy variable for the regional government (regions may be governed by a left- or right-leaning coalition), per capita gross domestic product (GDP), proportion of people >65 years, number of pharmacists and number of general practitioners. We included the same variables (with the exception of pharmacists and physicians, as the relevant data were incomplete). Because of possible age-related differences in drug use, we added the paediatric population; we also included the political cycle (in pre-election periods, cost-containment actions may be relaxed to increase consensus) and added a double control for time: first, we included a time dummy for every single month (162 variables) in the model; and second, we controlled for repeated seasonal effects (Fig. 2) through dummies identifying the quarter (three variables). These two controls have different meanings: the second ne captures seasonal trends in the pharmaceutical market that are repeated every year, particularly evident from Fig. 2, while the first aims at reducing endogeneity by capturing any contingent effect happening in a particular month that is not explicitly accounted for in our model. In fact, despite the important seasonal effects, every period can carry some peculiarity that is outside our model and can influence our dependent and explanatory variables. However, including monthly dummies alone would have been enough to explicitly capture seasonal effects. Moreover, both monthly (unique) dummies and seasonal (repeated every year) ones are significant and do not present collinearity issues. We further considered two other possible sources of endogeneity: turnaround plans and spillover effects. Since 2007, regions with important health-care deficits have been asked to implement a turnaround plan, which increases the pressure to adopt cost-containment measures. The effects of pharmaceutical policies would likely be artificially inflated if turnaround plans were not considered. Accordingly, we included three dummy variables related to turnaround plans: (1) whether the region was treated with a turnaround (*R* turnaround = 1 if the region has introduced a turnaround); (2) whether the turnaround was active (*T* turnaround = 1 after turnaround plan approval); and (3) a DD estimator of the turnaround plan’s effect (Turnaround = 1 if the region was treated and the policy was active). Second, regions may experience imitative pressure to adopt drug copayment. This tendency was captured by a variable that considers, for each month, the overall number of regions with drug copayments (Fig. 1). Our hypothesis is that this imitative pressure may strengthen negative and positive impacts of copayment on public and private expenditures, respectively, by reducing reaction time from prescribers and patients who have observed other regions. We hypothesise that this effect will only influence copayment, as copayment is the policy most discussed and best known by the population; the other two policies are primarily known by technical staff, making spillover effects less likely. We also tested a model with spillover effects on the other two policies, but their coefficients were never significant, so we removed them to create the most parsimonious models.

**Table 1:** Regional policies

Regions	Copayment		Prescription quotas		Therapeutic reference pricing	
	Years	Months of activation (number)	Years	Months of activation (number)	Years	Months of activation (number)
Piemonte	2002–2014	151		0		0
Valle d’Aosta		0	2006–2014	104		0
Lombardia	2002–2014	143		0		0
PA Bolzano	2002–2014	149		0		0
PA Trento		0		0		0
Veneto	2003–2014	139		0		0
Friuli Venezia Giulia		0		0		0
Liguria	2002–2014	144	2007–2014	94	2007–2014	94
Emilia Romagna	2011–2014	38		0		0
Toscana	2012–2014	26	2007–2014	93		0
Umbria	2011–2014	38		0		0
Marche		0	2007–2014	93		0
Lazio	2002–2005, 2008–2014	118	2007–2014	91		0
Abruzzo	2002–2014	149	2005–2014	113	2007–2014	94
Molise	2002–2014	144	2008–2014	73	2007–2014	91
Campania	2007–2014	94	2007–2014	88	2007–2014	88
Puglia	2002–2014	147		0	2007–2014	94
Basilicata	2013–2014	16	2007–2014	90	2007–2014	90
Calabria	2002–2005, 2009–2014	108	2012–2014	32	2007–2014	93
Sicilia	2004–2014	124	2007–2014	91	2007–2014	91
Sardegna	2002–2004	21	2012–2014	31	2007–2014	93

**Table 2:** Variables, measures and sources

Variable	Measure	Source
Per capita public pharmaceutical expenditures (ln)	ln of per capita monthly public pharmaceutical expenditures	Pharmaceutical observatory, CERGAS Bocconi
Per capita private pharmaceutical expenditures (ln)	ln of per capita monthly private pharmaceutical expenditures	Pharmaceutical observatory, CERGAS Bocconi
Per capita volumes of reimbursed drugs (ln)	ln of per capita monthly volumes (units) sold of reimbursed drugs	Pharmaceutical observatory, CERGAS Bocconi
Per capita volumes of nonreimbursed drugs (ln)	ln of per capita monthly volumes (units) sold of nonreimbursed drugs	Pharmaceutical observatory, CERGAS Bocconi
Copayment, prescription quotas and TRP	Dummy variables: 1 if the policy is active in the region during a focal month	Pharmaceutical observatory, CERGAS Bocconi
Region with turnaround plan	Dummy variable: 1 if a turnaround plan is active in the region during a focal month	Ministry of Health and OASI observatory
Average monthly income (×1000 euros)	Total regional monthly income per capita measured in 1000 euros (derived by a proportion of the annual income)	IISTAT
Share of >65-year-olds	Percentage of population >65 years (yearly average)	ISTAT
Share of <14 year-olds	Percentage of population <14 years (yearly average)	ISTAT
Number of regions with copayment	Number of regions, excluding the focal one, that have already introduced copayment policies during a focal month	Pharmaceutical observatory, CERGAS Bocconi
Left-leaning regional government	Dummy variable: 1 if the region has a left-leaning government during a focal month	Ministry of Internal Affairs
Pre-electoral period	Dummy variable: 1 if an election for the regional government occurs within 3 months from the focal month	Ministry of Internal Affairs
Observations	3423	
Number of regions and autonomous provinces	21	

*In*, * TRP* therapeutic reference pricing; * OASI*, * ISTAT* Italian Institute of Statistics, *ln (L N - NOT I N )* natural logarithm, *OASI* osservatorio sulle aziende e il sistema sanitario italiano, *CERGAS* centro di ricerca sulla gestione dell'assistenza sanitaria e sociale

### The model

A DD model was adopted and enriched to better address the parallel trend assumption (i.e. in the absence of the policy, dependent variables would vary similarly over time in both the treatment and control groups). In addition to standard procedures for estimating a DD effect, we adopted two strategies to better cope with the assumption. First, we controlled for the simultaneous presence of more than one policy at any time in every region, with the inclusion of interactions. Second, we included the difference between short- and long-term effects. This way of addressing the parallel trend assumption does not remove it but allows a more precise estimation of such a trend. The consequence of this strategy can be seen in the different *R* and *T* coefficients (see below) for copayment after inclusion of the other two policies. The model is described by the following equation:

1 $$y_{jt} = \beta_{0} + R_{j} \beta_{1} + T_{t} \beta_{2} + \beta_{3} D_{jt} + {\text{Run}}_{jt} \beta_{4} + \beta_{5} {\text{Interactions}}_{jt} + X_{jt} \beta_{6} + u_{jt} ,$$

where *R*_*j*_ = 1 if the region belongs to the treatment group, i.e. to the group of regions that have activated the policy for at least 1 month over the observed range; *T*_*t*_ = 1 in the treatment period (*T*_*t*_ = 0 before *t,*, the month when first the region introduces the focal policy); *D*_*jt*_ indicates that region *j* is on treatment at time *t*, i.e. region *j* belongs to the *R* = 1 group, *t* ≥ *t* and the policy has been introduced in region *j*; *β*_3_ is the main parameter of interest (DD); Run_*jt*_ is a pair of dummy variables that indicates whether the focal policy has been applied for <6 months or for at least 6 months (we tested different definitions of long term, finding consistent results and an indication that most differences between short- and long-term effects are visible using the 6-months split tables available from the authors); ${\text{Interactions}}_{jt}$ is the set of interactions among difference-in-differences in the three policies; and *X*_*jt*_ is the control variable matrix, including both quarterly (seasonal) dummies repeated every year and 162 monthly dummies.

The behavioural mediation hypothesis was tested with the Sobel–Goodman test and completed by a bootstrap estimation. The Sobel test, also known as the delta method, was introduced in 1982 [23] as a test of the significance of the indirect (mediated) effect. If *a* is the path from the independent variable and the mediator, and *b* is the path from the mediator to the dependent variable, then *a* × *b* is the indirect effect. After dividing the indirect effect by the square root of the variance *b*^2^ × standard error of *a* + *a*^2^ × standard error of b, a *Z* test is made on this ratio. When its value is >1.96, the mediation hypothesis is supported. However, MacKinnon et al. [24] provided evidence of the conservative nature of the Sobel test, which is, therefore, not very powerful. The main reason for the test being conservative is that the sampling distribution of the indirect effect is highly skewed. When the patter is positive, there is positive skew with many small estimates of the indirect effect and few very large ones. Since the Sobel test uses a normal approximation that presumes a symmetric distribution, it falsely presumes symmetry, which leads to a conservative test. For this reason, the Sobel test is generally associated with bootstrapping [25, 26], a nonparametric method based on resampling with replacement, which is done many times (1000 in this study), where the indirect effect is estimated at every replication. We estimated the indirect effect (and direct and total effects) using both the standard Sobel–Goodman test and the bootstrap method.

To consider possible simultaneous effects on private expenditures and volumes, we tested two mediation mechanisms. In other words, we allowed only volumes or private expenditures to react to policies in each model so we could establish their individual impacts. In particular, in the first model, we used public expenditures as the dependent variable, policies as independent variables and volumes as a mediator that was conditioned on all other control variables and private expenditures. In the second model, we used private expenditures as a mediator that was conditioned on all other control variables and volumes.

## Results

Figure 1 shows the number of regions that adopted each policy over the observed period (178 months), and monthly public and private expenditures are presented in Fig. 2 (a seasonal trend is evident). Copayment was introduced for the first time at month 24 (January 2002). At the end of the period (December 2010), copayment was present in 12 regions. Prescription quotas were first adopted at month 66 (May 2005) and were eventually implemented by nine regions. TRP was introduced by three regions at month 85 (January 2007) and eventually adopted by six other regions. Regions were not allowed to introduce TRP after January 2008, but regions in which the policy had already been introduced were allowed to maintain it.

### DD models for individual and interactive effects

Descriptive statistics are summarised in Table 3. Box 1 illustrates the model matrix. Results are illustrated in Table 4a (expenditures models) and Table 4b (volumes models). Box 1 illustrates the model matrix (Table 3).

**Box 1:** Model matrix

	Copayment only	Copayment + control variables	All three policies + control variables
Table 4a (expenditures)	Models 1 and 4	Models 2 and 5	Models 3 and 6
Table 4b (volumes)	Models 7 and 10	Models 8 and 11	Models 9 and 12

The two models in each cell refer to public and private drug expenditures and volumes of partially or fully reimbursed and nonreimbursed drugs

**Table 3:** Descriptive statistics

Variable	Mean	Standard deviation	Max	Min
Per capita public pharmaceutical expenditures (€)	15.63	3.02	28.10	7.48
Per capita private pharmaceutical expenditures (€)	9.73	2.38	20.11	2.32
Per capita volumes of reimbursed drugs (units)	1.57	0.28	3.15	0.83
Per capita volumes of nonreimbursed drugs (U)	0.85	0.17	1.43	0.50
Per capita public pharmaceutical expenditures	2.73	0.20	3.34	2.01
Per capita private pharmaceutical expenditures (ln)	2.24	0.26	3.00	0.84
Per capita volumes of reimbursed drugs (ln)	0.43	0.18	1.15	−0.19
Per capita volumes of nonreimbursed drugs (ln)	−0.18	0.20	0.36	−0.70
Copayment	0.50	0.50	1.00	0.00
Prescription quotas	0.28	0.45	1.00	0.00
TRP	0.24	0.43	1.00	0.00
Turnaround	0.21	0.40	1.00	0.00
Average monthly income (×1000 euros)	2.06	0.52	3.22	1.12
Share of >65 years	12.41	9.21	26.00	0.15
Share of <14 years	8.53	6.32	18.75	0.11
Number of regions with copayment	10.54	4.06	16.00	0.00
Left-leaning regional government	0.57	0.49	1.00	0.00
Pre-electoral period	0.05	0.22	1.00	0.00

*TRP* therapeutic reference pricing,* In*

**Table 4:** Models on ln of (a) expenditures, (b) volumes (sold units)

Variables	(1)	(2)	(3)	(4)	(5)	(6)
	Public expenditures	Public expenditures	Public expenditures	Private expenditures	Private expenditures	Private expenditures
(a)						
Copayment	−0.136***	−0.069***	−0.060***	0.103***	0.079***	0.090***
Copayment (>6 m)	−0.060***	0.011*	0.014**	0.135***	−0.017*	−0.016
Prescription quotas			0.001			0.061***
Prescription quotas (>6 m)			−0.018**			0.003
TRP			−0.013			0.024
TRP (> 6 m)			0.037***			0.069***
Copayment × prescription quotas			−0.049***			−0.009
Copayment × TRP			0.007			−0.040***
Prescription quotas × TRP			0.019**			−0.021*
*R* (copayment)	0.205***	−0.038	0.014	−0.149	−0.029	0.039
*R* (prescription quotas)			0.092			0.032
*R* (TRP)			−0.307***			−0.425***
*T* (copayment)	0.004	0.230	0.350*	0.193***	0.140	0.207
*T* (prescription quotas)			−0.126***			−0.080
*T* (TRP)			0.191			0.238
Region with turnaround plan		−0.022***	−0.014***		0.002	−0.015**
*R* (turnaround)		0.043	0.180***		−0.157**	0.098**
*T* (turnaround)		0.191	–		0.122	
Average monthly income (×1000)		−0.313***	−0.336***		−0.195***	−0.176***
Share of >65 year		0.009***	0.008***		0.012***	0.015***
Share of <14 year		0.009***	0.011***		−0.033***	−0.025***
Number of regions with copayment		−0.017	−0.015		−0.001	−0.001
Left-leaning regional government		−0.000	0.002		−0.035***	−0.028***
Pre-electoral period		−0.005	−0.005		0.009	0.010
Monthly and quarterly dummy variables	No	Yes	Yes	No	Yes	Yes
Constant	2.657***	3.105***	3.106***	2.069***	2.740***	2.515***
Observations	3486	3423	3423	3486	3423	3423
Number of regions	21	21	21	21	21	21
*R* ^2^ (overall)	0.104	0.662	0.753	0.160	0.416	0.674
*R* ^2^ (within)	0.191	0.869	0.875	0.285	0.833	0.842
*R* ^2^ (between)	0.038	0.511	0.633	0.009	0.032	0.368
*χ* ^2^	814.1	21,242	22,583	1377	15,907	17,030
*p* value (*χ* ^2^)	0.000	0.000	0.000	0.000	0.000	0.000

Variables	(7)	(8)	(9)	(10)	(11)	(12)
	Class-A units	Class-A units	Class-A units	Other classes	Other classes	Other classes
(b)						
Co-payment	−0.038***	−0.033***	−0.018***	−0.009	−0.002	0.007
Co-payment (>6 m)	0.169***	0.004	0.009	−0.076***	−0.001	−0.002
Prescription quotas			0.022***			0.004
Prescription quotas (>6 m)			−0.005			0.002
TRP			0.025**			0.055***
TRP (>6 m)			0.034***			0.016*
Co-payment × Prescription quotas			−0.046***			0.005
Co-payment × TRP			−0.032***			−0.036***
Prescription quotas × TRP			0.014*			−0.003
*R* (co-payment)	0.003	0.016	0.056	−0.040	−0.050	−0.004
*R* (prescription quotas)			0.097***			0.055
*R* (TRP)			−0.196***			−0.279***
*T* (co-payment)	−0.001	−0.195	−0.219	−0.152***	0.152	0.462***
*T* (prescription quotas)			−0.002			−0.321***
*T* (TRP)			0.007			0.199*
Region with turnaround plan		−0.006	−0.000		0.002	−0.007*
*R* (turnaround)		0.019	0.096**		−0.058	0.099**
*T* (turnaround)		0.014	–		0.125	
Average monthly income (×1000)		−0.183***	−0.171***		0.005	0.029
Share of >65 year		0.001	0.001		0.000	0.002*
Share of <14 year		−0.012***	−0.009***		−0.016***	−0.011***
Number of regions with co-payment		0.022	0.024		−0.046***	−0.046***
Left-leaning regional government		0.001	0.008**		0.001	0.007**
Pre-electoral period		−0.001	−0.000		−0.008*	−0.008*
Monthly and quaterly dummy variables	No	Yes	Yes	No	Yes	Yes
Constant	0.365***	0.817***	0.727***	0.049	0.276***	0.108
Observations	3234	3171	3171	3234	3171	3171
Number of regions	21	21	21	21	21	21
*R* ^2^ (overall)	0.030	0.663	0.748	0.049	0.442	0.642
*R* ^2^ (within)	0.105	0.863	0.871	0.066	0.845	0.854
*R* ^2^ (between)	0.001	0.397	0.568	0.041	0.227	0.521
*χ* ^2^	370.4	18,755	20,043	228.7	16,256	17,232
*p* value (*χ* ^2^)	0.000	0.000	0.000	0.000	0.000	0.000

Class-A stands for reimbursable drugs

*** *p* < 0.01; ** *p* < 0.05, * *p* < 0.1

#### Effects on public expenditures

Copayment (model 3) directly reduced public expenditures by 6 % in the short term and 4.6 % (6 % in the short term + 1.4 % after the first 6 months) in the long term. When prescription quotas were also activated, the negative impact of copayment increased by 4.9 %, while the interaction between copayment and TRP was not significant. Interestingly, TRP had no effect in the short term, while after the first 6 months, its presence increased public expenditures by 3.7 %. As expected, prescription quotas showed their direct effect on public expenditures in the long term (−1.8 %), as their mechanism of transmission is thought to be primarily behavioural and is not thought to be mediated by elasticity effects. However, the interaction between prescription quotas and TRP is positive, leading to a further increase in public expenditures due to TRP (+1.9 %).

#### Effects on volumes

Volumes of reimbursed drugs (model 9) were affected by copayments in a similar but smoother manner compared with public expenditures, with a 1.8 % short-term decrease and a 0.9 % long-term decrease (−1.8 % in the short term + 0.9 % after the first 6 months); however, the long-term effect was not significant. The interaction of copayment and prescription quotas contributed to an additional 4.6 % decline in volumes. The interaction between copayment and TRP was significant and negative also, with an additional effect of −3.2 %. These reductions are partly compensated for by the fact that the individual effect of prescription quotas and TRP were positive (+2.2 and +2.5 %, respectively, with TRP also increasing it effects in the long run by an additional 3.4 %). In other words, when not coupled with a copayment, the other two policies cause an increase in volumes; however, when copayment is active, their impact is reduced (TRP) or even reversed (prescription quotas). Interestingly, TRP is also associated with an increase in consumption of nonreimbursed drugs (+5.5 if cost sharing is not active, +1.9 % otherwise; model 12). If perfect substitution among products within the same therapeutic class is assumed, TRP represents a perceived reduction in prices for prescribers and patients and should increase volumes. The increase in volumes of nonreimbursed drugs by 5.5 %, may have been the result of a perception of substitutability of products under TRP. This also signals that private expenditures might be endogenous to TRP, and—as confirmed by mediation analysis—where private expenditures will be explicitly controlled for.

#### Effects on patient expenditures

**Fig. 3:**
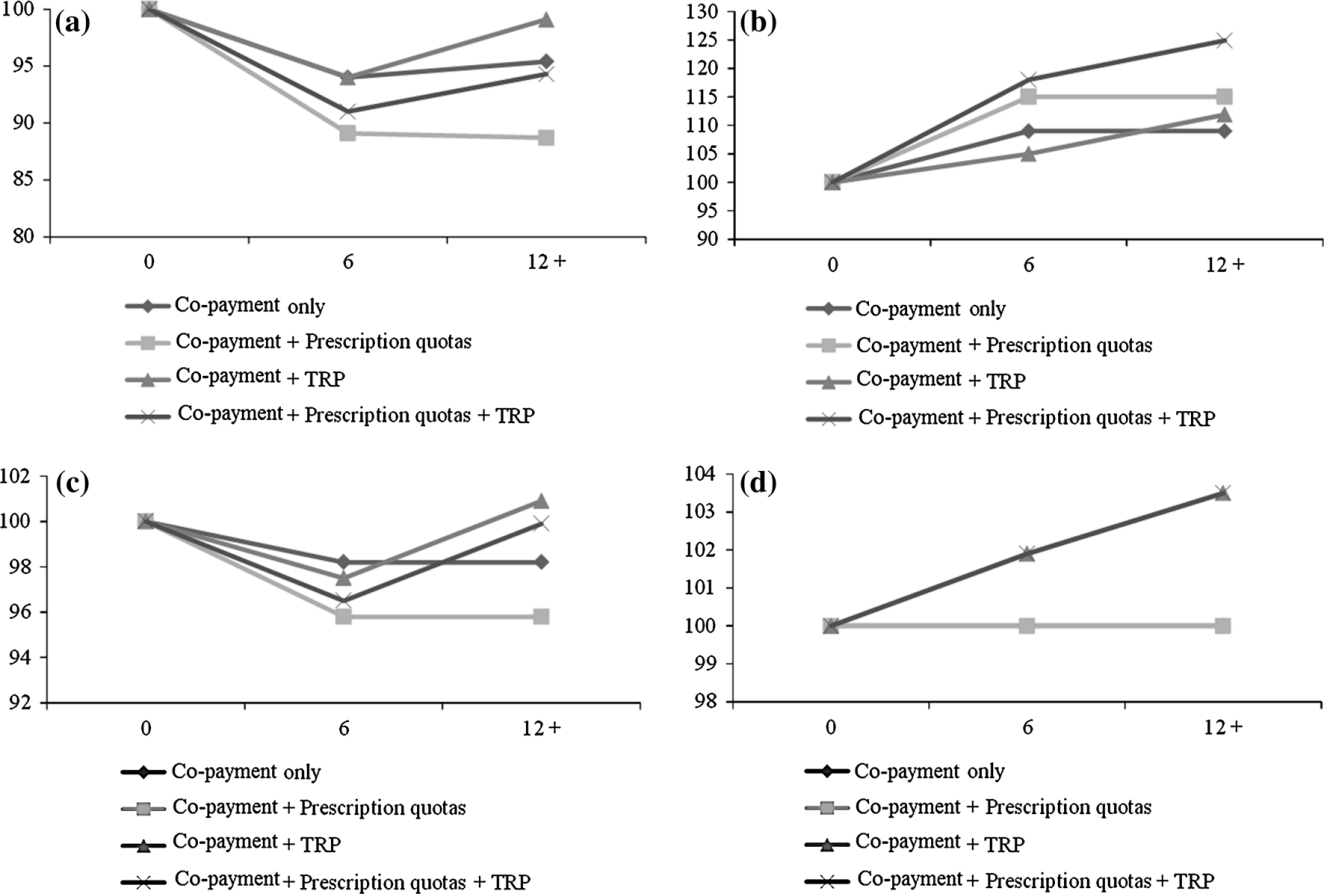
Simultaneous impact of pharmaceutical policies over time on public expenditures (**a**), private expenditures (**b**), units of (partially and fully) reimbursed drugs (**c**), and units of nonreimbursable drugs (**d**). Initial levels are set to 100. Simulations were performed using models 3, 6, 9 and 12

Dynamics of public expenditures and the contemporaneous impact on volumes help explain the effect of the three policies on private expenditures (model 6). First, the short-term effect of copayment on private expenditure (+9 %) mirrors the decrease in public expenditures (−6 %, model 3). To evaluate the meaning of the two effects in absolute terms, these two difference-in-differences coefficients were applied to the average prepolicy levels of expenditures reported by treated regions. In absolute terms, applying estimated coefficients to the average prepolicy level of expenditures, the estimated decrease in public expenditures is 1.05 euros per capita/month, while the increase in private expenditures is 0.64 euros per capita/month: the difference of 0.41 euros per capita/month is due to reduced volumes (−1.8 %). The opposite long-term effect of cost sharing on public expenditures is reflected in a similar increase in private expenditures, even though the coefficient is not significant. Second, while prescription quotas have no effects on public expenditures in the short term, since the impact on volumes is positive, the effect on patient expenditure is also positive (+6.1 %). Therefore, prescription quotas increase the consumption of reimbursed drugs rather than creating a simple reallocation of prescriptions on different products; however, expenditures increases for patients only, signalling that physicians tend to prescribe such drugs without activating public reimbursement (patients can privately buy reimbursement drugs with a prescription written by a physician on a signed paper and not on the official prescription document). Figure 3 summarises the effects.

Some control variables also had an influence on drugs expenditures. For example, regions with larger proportions of elderly and young patients showed a higher level of public expenditures, while patient expenditure was higher in regions with older populations and lower in regions with younger ones. Volumes of reimbursed drugs were lower in regions with larger young populations, while both the young and the elderly tended to consume fewer nonreimbursed drugs. Income was negatively correlated with both public and private expenditures and with volumes of reimbursed drugs. This effect contradicts the expectation that the most affluent patients may be more willing to shift from public to private expenditures [18], and in the case of exemptions based on income, they are obliged to contribute more. However, because of copayments, patients not eligible for exemptions (i.e. the most affluent) must also pay for reimbursed drugs, which creates less demand and, consequently, a lower level of public and private expenditures. The same phenomenon has been reported by the Italian Department of Health with respect to specialist visits, where patients without exemptions reduced their demand after activation of cost-sharing, as opposed to patients with exemptions based on income, whose demand remained stable.

Turnaround plans appear to be an important control variable. Regions with such plans include those spending more for drugs and experiencing the highest decrease in public and private drug expenditures. As expected, private drug expenditures in regions governed by left-leaning coalitions were lower than regions governed by right-leaning coalitions. In fact, left-leaning coalitions appeared to be more sensitive to social issues and public coverage (volumes of reimbursed drugs are higher in these regions). The political cycle had no important influence on drug expenditures.

### Test of a mediation by behavioural mechanisms

Copayment is the only policy in which its relationship with public expenditures is mediated by both volume and private expenditure. Prescription quotas and TRP may involve transmission mechanisms other than volume, such as change in mix of prescribed drugs, but these data were not available. Results of mediation tests are presented in Table 5, and details are provided in Table 6.

**Table 5:** Mediation tests

Independent variable	Mediator	Support for mediation hypthesis	Effect mediated	Direct effect on public expenditures	Indirect effect on public expenditures	Total effect on public expenditures	Sobel–Goodman test (*p* value)
Cost sharing	Volumes	Yes	59.7 %	−3.0 %	−4.4 %	−7.4 %	<0.01
	Private expenditures	Yes	26.5 %	−3.0 %	−1.1 %	−4.1 %	<0.01
Prescription quotas	Volumes	Yes (only indirect effect)	161.0 %	NS	2.0 %	NS	<0.05
	Private expenditures	No	35.5 %	NS	NS	NS	>0.05
TRP	Volumes	No	NS	NS	NS	NS	>0.05
	Volumes (in the long run)	Yes	−152.2 %	3.6 %	−2.2 %	NS	<0.01
	Private expenditures	No	NS	NS	NS	NS	>0.1

*TRP* therapeutic reference pricing,* NS* not significant

The first copayment mediation model , with drug volumes as the mediator, supports the mediation hypothesis. By holding private expenditures constant, copayment reduces volumes by 5.4 %. The elasticity of public expenditures to volumes is 0.81, such that a 5.4 % reduction in volumes produces a 4.4 % decline in public spending. Therefore, the direct impact of copayment (−3 %) is reinforced by the indirect effect, and the total effect of −7.4 % is partly mediated (59.7 % of the total effect) by a volume effect. We also assessed the amount of burden transferred to patients by holding volumes constant, thus observing the mediation of private expenditures. The total effect of copayment on public expenditures was reduced from 7.4 to 4.1 % when volumes were held constant, whereas the direct effect did not change (−2.9 %). In this case, copayment produced a 7.5 % increase in private expenditures, and the elasticity of substitution between private and public expenditures was −0.14. Therefore, the indirect effect of copayment via private expenditures was −1.1 %, mediating 26.5 % of the total effect on public expenditures. No mediation was observed in the long term, so we removed the long-term effect from Table 5. Comparing the two tests on copayment, we provide evidence that a behavioural volume effect attenuated the burden of a shift from public to private coverage. The effects of prescription quotas were not mediated by private expenditures. However, the effects of prescription quotas were fully mediated by volumes, creating an increase of 2.5 %. Such an increase led to a +2.0 % indirect effect of prescription quotas on public expenditures. Finally, the effect of TRP was only mediated by volumes in the long run. Differently from what emerged from regression analysis, when holding private expenditures constant, the impact of TRP on volumes was negative rather than positive. In fact, controlling for private expenditures, TRP produced a decrease in volumes of 2.7 %, which translates into a 2.2 % reduction in public expenditures. The direct effect, instead, remained positive (3.7 %), and therefore the total effect was not significantly different from 0. This situation is known as inconsistent mediation, because the direct effect has an opposite sign compared with the indirect effect, resulting in a null total effect. In other words, the mediated effect compensates for the direct one. Bootstrap estimations were consistent and confirmed the Sobel–Goodman test results (Table 6). All reverse causality tests rejected mediation at the 95 % confidence level.

**Table 6:** Results of the Sobel–Goodman test and bootstrap analysis

	Effects	Coefficient	Standard error	*Z*	Significance	Bootstrap coefficient
DV: public expentitures; IV: cost sharing; MV: volumes						
Sobel		−0.044	0.007	−6.453	***	
Goodman-1 (Aroian)		−0.044	0.007	−6.452	***	
Goodman-2		−0.044	0.007	−6.454	***	
a coefficient		−0.055	0.008	−6.495	***	
b coefficient		0.810	0.014	56.677	***	
Indirect effect		−0.044	0.007	−6.453	***	−0.044
Direct effect		−0.030	0.007	−4.504	***	−0.030
Total effect		−0.074	0.010	−7.823	***	−0.074
Proportion of total effect mediated	59.7 %					
Ratio of indirect to direct effect	1.482					
Ratio of total to direct effect	2.482					
DV: public expentitures; IV: cost sharing; MV: private expenditures						
Sobel		−0.011	0.002	−5.342	***	
Goodman-1 (Aroian)		−0.011	0.002	−5.333	***	
Goodman-2		−0.011	0.002	−5.352	***	
Coefficient^a^		0.076	0.013	5.679	***	
Coefficient^b^		−0.143	0.009	−15.742	***	
Indirect effect		−0.011	0.002	−5.342	***	−0.011
Direct effect		−0.030	0.007	−4.504	***	−0.030
Total effect		−0.041	0.007	−5.922	***	−0.041
Proportion of total effect mediated	26.5 %					
Ratio of indirect to direct effect	0.360					
Ratio of total to direct effect	1.360					
DV: public expentitures; IV: prescription quotas; MV: volumes						
Sobel		0.020	0.009	2.231	**	
Goodman-1 (Aroian)		0.020	0.009	2.23	**	
Goodman-2		0.020	0.009	2.231	**	
Coefficient^a^		0.025	0.011	2.232	**	
Coefficient^b^		0.810	0.014	56.677	***	
Indirect effect		0.020	0.009	2.231	**	0.020
Direct effect		−0.008	0.009	−0.874		−0.008
Total effect		0.012	0.012	0.997		0.012
Proportion of total effect mediated	161.0 %					
Ratio of indirect to direct effect	−2.639					
Ratio of total to direct effect	−1.639					
DV: public expentitures; IV: prescription quotas; MV: private expenditures						
Sobel		−0.004	0.002	−1.665	*	
Goodman-1 (Aroian)		−0.004	0.002	−1.661	*	
Goodman-2		−0.004	0.002	−1.668	*	
Coefficient^a^		0.029	0.017	1.674	*	
Coefficient^b^		−0.143	0.009	−15.742	***	
Indirect effect		−0.004	0.002	−1.665	*	−0.004
Direct effect		−0.008	0.009	−0.874		−0.008
Total effect		−0.012	0.009	−1.303		−0.012
Proportion of total effect mediated	35.5 %					
Ratio of indirect to direct effect	0.550					
Ratio of total to direct effect	1.550					
DV: public expentitures; IV: TRP; MV: volumes						
Sobel		−0.020	0.012	−1.769	*	
Goodman-1 (Aroian)		−0.020	0.012	−1.769	*	
Goodman-2		−0.020	0.012	−1.769	*	
Coefficient^a^		−0.025	0.014	−1.770	*	
Coefficient^b^		0.810	0.014	56.677	***	
Indirect effect		−0.020	0.012	−1.769	*	−0.020
Direct effect		0.010	0.011	0.905		0.010
Total effect		−0.010	0.016	−0.643		−0.010
Proportion of total effectmediated	197.9 %					
Ratio of indirect to direct effect	−2.021					
Ratio of total to direct effect	−1.021					
DV: public expentitures; IV: TRP (long run); MV: volumes						
Sobel		−0.022	0.007	−3.241	***	
Goodman-1 (Aroian)		−0.022	0.007	−3.241	***	
Goodman-2		−0.022	0.007	−3.242	***	
Coefficient^a^		−0.027	0.008	−3.246	***	
Coefficient^b^		0.810	0.014	56.679	***	
Indirect effect		−0.022	0.007	−3.241	***	−0.022
Direct effect		0.036	0.007	5.556	***	0.036
Total effect		0.014	0.009	1.534		0.014
Proportion of total effect mediated	−152.2 %					
Ratio of indirect to direct effect	−0.604					
Ratio of total to direct effect	0.396					
DV: public expentitures; IV: TRP; MV: private expenditures						
Sobel		−0.003	0.003	−0.902		
Goodman-1 (Aroian)		−0.003	0.003	−0.9		
Goodman-2		−0.003	0.003	−0.904		
Coefficient^a^		0.020	0.022	0.904		
Coefficient^b^		−0.143	0.009	−15.742	***	
Indirect effect		−0.003	0.003	−0.902		−0.003
Direct effect		0.010	0.011	0.905		0.010
Total effect		0.007	0.012	0.621		0.007
Proportion of total effect mediated	−40.2 %					
Ratio of indirect to direct effect	−0.287					
Ratio of total to direct effect	0.713					

*DV* dependent variable,* IV* independent variable, *MV* mediator variable, * TRP* therapeutic reference pricing

^***^p < 0.01; ^**^ p < 0.05, ^*^ p < 0.1

## Discussion

This paper discusses the effects of pharmaceutical policies, simultaneously and variously implemented, on retail drug expenses and volumes and considers possible behavioural transmission mechanisms. These two topics have not been previously investigated.

Italy is the ideal country for assessing the simultaneous effects of policies, as policies have been applied in different regions at different times. Italy’s regional copayment is unique within the EU, but in most countries, prescribing policies (clinical governance and prescription targets) are implemented at regional and local levels. Hence, this analysis can be largely extended to other countries. The model was designed to:

 (1) Estimate the simultaneous impact of different policies in the short and long term.

(2) Control for the simultaneous presence of other policies, thus addressing the parallel trend problem.

(3) Incorporate and model endogeneity issues, such as turnaround plans and possible spillover effects.

(4) Assess the direct and indirect (or mediated) impact of policies.

The first interesting result of our study is that combined policies do not necessarily produce a higher impact than individual policies. For example, when copayment and prescription quotas are combined, the final impact is higher than in the case of each policy being implemented independently; the impact of a combination of former policies with TRP is counterintuitive instead; Second, we generally observed a larger impact of policies in the short term, as the trend was often reversed in the long term, although not sufficiently to compensate the final impact, which was usually in the expected direction. Third, analysis of mediation shows that the negative impact of copayment on public expenditures is primarily caused by volumes, whereas the shift from public to private expenditures is less important. The demand for prescription-only drugs appears to be price-elastic. The TRP transmission mechanism is driven by an expenditure shift in the short term and by volumes in the long term, with a final unpredicted impact on public expenditures that increases instead of being controlled.

This study has some limitations. First, we used aggregate data to estimate the simultaneous effects of the policies. Some policies are disease specific (e.g. prescription quotas and TRP), and their effects would have been better captured by more granular data. Further research is needed on this topic, but the scope of this study was intentionally broad. Second, we assumed that copayment was equal across regions, but Fiorio and Siciliani [5] have shown that the effect of copayment also depends on fee per prescription. In this study we provide an average effect of copayment. We acknowledge that it may vary in size (but not in direction) according to the different fees per prescription. Finally, we were unable to fully disentangle prescription and consumption, as we only observed expenditures for drugs that were prescribed and sold. In other words, even though we observed changes in volumes due to a joint decision of patient and general practitioner, we could not shed more light on the agency relationship between the two actors, e.g. when a drug is prescribed by the practitioner but not bought by the patient.

## Conclusions

Evidence regarding the impact over time of regional policies diversely introduced at different times have important policy implications. First, pharmaceutical policies interact with each other, and the combined effect may be different from what would be expected from the sum of each individual policy. Hence, policymakers should be very careful when designing mixed policies due to their unexpected combined effects. Second, the impact of policies tends to reduce over time. If longer-term impact is desired, it would be appropriate to introduce some changes over time (e.g. increasing copayment or reducing exemption from copayment). Third, policies have multiple effects that should be considered when they are designed. For example copayments may be intended to reduce volumes, because they are considered inappropriate, and/or to partially shift the burden of drug expenditures from third-party payers to patients. Our analysis shows that the impact on volumes is more important than a coverage shift in decreasing public drug expenditure. Finally, pharmaceutical policies may have an unintended impact on health and health care. Copayment is applied where per capita drug volumes are low. If lower volumes are associated with appropriate drug usage, a further decrease in volumes may imply undertreatment, with an important impact on health and health expenditures. However, because many drugs analysed in this study are prescribed by general practitioners for chronic diseases (e.g. hypertension, diabetes, hypercholesterolaemia), undertreatment has a long-term impact on health and health-care expenditures, a time frame that tends not to be considered by payers.

Hence, despite its limitations, this study contributes to research and policy-making decisions by presenting a detailed and behavioural perspective on policy impacts, as we adopted a multifaceted (patients, prescribers, policymakers) perspective regarding pharmaceutical policies.

## References

[1] Olson MK (1995). The effects of UK pharmaceutical policy on government drug expenditure: cost control and incentives for R&D. Int. J. Econ. Bus..

[2] Atella V (1999). Drug cost containment policies in Italy: are they really effective in the long-run?-The case of minimum reference price. Health Policy.

[3] Buzzelli C, Kangasharju A, Linnosmaa I, Valtonen H (2006). Impact of generic substitution on pharmaceutical prices and expenditures in OECD countries. J. Pharm. Fin. Econ. Policy.

[4] Jommi C, Armeni P, De Luca C, Otto M, Vella V, Cantù E (2011). Il governo regionale dell’assistenza farmaceutica e il suo impatto sulla spesa. L’aziendalizzazione della sanità in Italia, Rapporto OASI.

[5] Fiorio CV, Siciliani L (2010). Copayments and the demand for pharmaceuticals: evidence from Italy. Econ. Model..

[6] Galizzi MM, Ghislandi S, Miraldo M (2011). Effects of reference pricing in pharmaceutical markets: a review. PharmacoEconomics.

[7] Ghislandi S, Armeni P, Jommi C (2013). The impact of generic reference pricing in Italy, a decade on. Eur. J. Health Econ..

[8] Fischer MA, Choudhry NK, Winkelmayer WC (2007). Impact of medicaid prior authorization on angiotensin-receptor blockers: can policy promote rational prescribing?. Health Aff..

[9] Li X, Anis AH (2013). Cost sharing of prescription drugs and demand for health-care utilization among seniors with rheumatoid arthritis. Appl. Econ. Lett..

[10] Skipper N (2012). On reimbursement reforms and stockpiling of prescription drugs: the case of insulin. Health Policy.

[11] Damiani G, Federico B, Anselmi A, Bianchi CB, Silvestrini G, Iodice L, Navarra P, Da Cas R, Raschetti R, Ricciardi W (2014). The impact of Regional copayment and National reimbursement criteria on statins use in Italy: an interrupted time-series analysis. BMC Health Serv. Res..

[12] Damiani G, Federico B, Silvestrini G, Bianchi CBNA, Anselmi A, Iodice L, Ronconi A, Navarra P, Da Cas R, Raschetti R (2013). Impact of regional copayment policy on selective serotonin reuptake inhibitor (SSRI) consumption and expenditure in Italy. Eur. J. Clin. Pharmacol..

[13] Noyce PR, Huttin C, Atella V, Brenner G, Haaijer-Ruskamp FM, Hedvall M-B, Mechtler R (2000). The cost of prescription medicines to patients. Health Policy.

[14] Ghislandi S, Krulichova I, Garattini L (2005). Pharmaceutical policy in Italy: towards a structural change?. Health Policy.

[15] Gemmill MC, Thomson S, Mossialos E (2008). What impact do prescription drug charges have on efficiency and equity? evidence from high-income countries. Int. J. Equity Health.

[16] Giuliani G, Selke G, Garattini L (1998). The German experience in reference pricing. Health Policy.

[17] Fattore G, Jommi C (2008). The last decade of italian pharmaceutical policy. Pharmacoeconomics.

[18] Osmed: L’uso dei farmaci in Italia—Rapporto OsMed 2012. In Monograph (2012)

[19] Jommi, C., Minghetti, P.: Pharmaceutical Pricing Policies in Italy. In: Babar, Zaheer-Ud-Din (eds.) Pharmaceutical prices in the 21st century, pp. 131–150. Springer, New York (2015)

[20] Jommi C, Costa E, Michelon A, Pisacane M, Scroccaro G (2013). Multi-tier drugs assessment in a decentralised health care system. The Italian case-study. Health Policy.

[21] Athey S, Imbens GW (2006). Identification and inference in nonlinear difference-in-differences models. Econometrica.

[22] Bertrand, M., Duflo, E., Mullainathan, S.: How Much Should We Trust Differences-in-Differences Estimates? National Bureau of Economic Research Working Paper Series No. 8841 (2002)

[23] Sobel ME (1982). Asymptotic confidence intervals for indirect effects in structural equation models. Sociol. Methodol..

[24] MacKinnon DP, Warsi G, Dwyer JH (1995). A simulation study of mediated effect measures. Multivar. Behav. Res..

[25] Bollen KA, Stine R (1990). Direct and indirect effects: classical and bootstrap estimates of variability. Sociol. Methodol..

[26] Shrout PE, Bolger N (2002). Mediation in experimental and nonexperimental studies: new procedures and recommendations. Psychol. Methods.

